# Hybrid, metal oxide-peptide amphiphile micelles for molecular magnetic resonance imaging of atherosclerosis

**DOI:** 10.1186/s12951-018-0420-8

**Published:** 2018-11-15

**Authors:** Christopher Poon, Juan Gallo, Johan Joo, Timothy Chang, Manuel Bañobre-López, Eun Ji Chung

**Affiliations:** 1 Department of Biomedical Engineering, University of Southern California, 1042 Downey Way, Los Angeles, CA 90089 USA; 2 Advanced (Magnetic) Theranostic Nanostructures Lab, Department of Life Sciences, International Iberian Nanotechnology Laboratory, Avenida Mestre José Veiga, Braga, Portugal; 3 Department of Materials Science and Chemical Engineering, University of Southern California, 925 Bloom Walk, Los Angeles, CA 90089 USA; 4 Eli and Edythe Broad Center for Regenerative Medicine and Stem Cell Research, Keck School of Medicine, University of Southern California, Los Angeles, CA USA; 5 Division of Nephrology and Hypertension, Department of Medicine, Keck School of Medicine, University of Southern California, Los Angeles, CA USA; 6 Norris Comprehensive Cancer Center, Keck School of Medicine, University of Southern California, Los Angeles, CA USA

**Keywords:** Hybrid nanoparticle, Micelle, Targeting, Magnetic resonance imaging, Atherosclerosis, Iron oxide, Manganese oxide

## Abstract

**Background:**

Atherosclerosis, a major source of cardiovascular disease, is asymptomatic for decades until the activation of thrombosis and the rupture of enlarged plaques, resulting in acute coronary syndromes and sudden cardiac arrest. Magnetic resonance imaging (MRI) is a noninvasive nuclear imaging technique to assess the degree of atherosclerotic plaque with high spatial resolution and excellent soft tissue contrast. However, MRI lacks sensitivity for preventive medicine, which limits the ability to observe the onset of vulnerable plaques. In this study, we engineered hybrid metal oxide-peptide amphiphile micelles (HMO-Ms) that combine an inorganic, magnetic iron oxide or manganese oxide inner core with organic, fibrin-targeting peptide amphiphiles, consisting of the sequence CREKA, for potential MRI imaging of thrombosis on atherosclerotic plaques.

**Results:**

Hybrid metal oxide-peptide amphiphile micelles, consisting of an iron oxide (Fe-Ms) or manganese oxide (Mn-Ms) core with CREKA peptides, were self-assembled into 20–30 nm spherical nanoparticles, as confirmed by dynamic light scattering and transmission electron microscopy. These hybrid nanoparticles were found to be biocompatible with human aortic endothelial cells in vitro, and HMO-Ms bound to human clots three to five times more efficiently than its non-targeted counterparts. Relaxivity studies showed ultra-high r_2_ value of 457 mM^−1^ s^−1^ and r_1_ value of 0.48 mM^−1^ s^−1^ for Fe-Ms and Mn-Ms, respectively. In vitro, MR imaging studies demonstrated the targeting capability of CREKA-functionalized hybrid nanoparticles with twofold enhancement of MR signals.

**Conclusion:**

This novel hybrid class of MR agents has potential as a non-invasive imaging method that specifically detects thrombosis during the pathogenesis of atherosclerosis.

**Electronic supplementary material:**

The online version of this article (10.1186/s12951-018-0420-8) contains supplementary material, which is available to authorized users.

## Background

Cardiovascular disease (CVD) is the leading cause of death in the United States, with over 800,000 deaths each year [1]. Atherosclerosis, which represents close to 70% of all CVDs, is a degenerative inflammatory disease that leads to the formation of thrombosis and increased build-up of rupture-prone or vulnerable plaques [2]. Its progression is often asymptomatic for decades until the onset of acute cardiovascular events, such as myocardial infarction or stroke, thereby making early detection of atherosclerosis difficult [3, 4]. Noninvasive nuclear imaging techniques, such as magnetic resonance imaging (MRI), have recently been used to diagnose coronary artery disease and preliminarily characterize plaque structure [5]. MRI has high spatial resolution and excellent soft tissue contrast, and does not require the use of ionizing radiation, making repetitive morphological follow-up possible [6, 7]. Nevertheless, MRI has limited sensitivity, making it difficult to differentiate and target diseased sites with conventional contrast agents.

Nanocarriers offer an alternative to conventional contrast agents and have the ability to carry a high content of contrast agents and target diseased areas due to the enhanced permeability and retention (EPR) effect characteristic to leaky endothelium and damaged vasculature found in atherosclerotic plaques [8–17]. A variety of inorganic nanomaterials (e.g. quantum dots, gold, carbon nanotubes, mesoporous silica metal oxide) have been investigated for cardiovascular diseases [18–20]. Specifically, iron oxide (Fe-NPs) and manganese oxide nanoparticles (Mn-NPs) are effective MRI contrast agents that have been used to image a wide variety of diseases [21–23]. Fe-NPs are negative contrast agents that reduce the transverse relaxation time (*T*_2_) to produce dark contrast-enhanced signals, while Mn-NPs are paramagnetic agents that shorten the longitudinal relaxation time (*T*_1_) of water proton to increase the bright signal intensity of a bright signal [24, 25]. However, most of these inorganic nanoparticles face problems with cellular toxicity and nonbiodegradability [18, 26, 27]. To mediate these issues, inorganic nanomaterials can be functionalized with organic components to improve colloidal stability, biocompatibility, biodegradability, and enhanced magnetic properties for imaging [28–31].

Previously, we have incorporated the fibrin-binding peptide, CREKA, into a class of organic nanoparticles called peptide amphiphile micelles (PAMs) for targeting thrombosis in atherosclerosis [32–35]. The CREKA micelles bind to the entire surface of the plaque, but concentrate at the shoulder portion of the plaque, a location that is prone to rupture and consist of microthrombi [36]. To combine the beneficial features of both organic and inorganic nanoparticles, in this work, we develop hybrid metal oxide PAMs (HMO-Ms) that consist of a highly crystalline iron or manganese oxide core encapsulated with organic, fibrin-targeting peptide amphiphiles for potential MRI detection of vulnerable plaques. HMO-Ms were self-assembled using a dry-film hydration method and found to be monodisperse, spherical nanoparticles of ~ 20–30 nm and stable in presence of serum. In vitro biocompatibility assays showed that these hybrid nanoparticles were nontoxic to human aortic endothelial cells (hAECs), and HMO-Ms demonstrated to have three to five times higher binding affinity than non-targeted PAMs on fibrin-containing clots derived from human plasma and thrombin. Moreover, the iron oxide- and manganese oxide-containing PAMs (Fe-Ms and Mn-Ms), exhibited enhanced *T*_2_- and *T*_1_-MRI-contrast, respectively. Our results highlight the potential of these inorganic/organic hybrid nanoparticles as highly effective delivery vehicles for contrast agents in MRI applications.

## Results and discussion

### Preparation and characterization of HMO-Ms

Metal oxide nanoparticles made of Fe_3_O_4_ (magnetite) were prepared following thermal decomposition protocols from iron acetylacetonate [37]. Transmission electron microscopy (TEM) showed spherical morphology with a diameter of 3.7 ± 0.8 nm (Additional file 1: Fig. S1A, B). Similarly, Mn-NP were also fabricated following thermal decomposition protocols using manganese oleate [38]. TEM confirmed Mn-NP were spherical in shape with a diameter of 11.1 ± 1.1 nm (Additional file 1: Fig. S2A, B). Fe and Mn presence in Fe-NP and Mn-NP, respectively, was corroborated by EDXS (Additional file 1: Figs. S1C, S2C). In both cases, nanoparticles were coated with oleate, which provided a lipid monolayer for functionalization with peptide amphiphiles (PAs).

To synthesize PAs, the fibrin binding peptide, CREKA, was conjugated onto DSPE-PEG(2000)-maleimide via thioether linkage (Additional file 1: Fig. S3). Using dry-film hydration methods, varying amount of Fe-NPs, from 0.05 μmol to 0.5 μmol, were modified with 50 nmol of DSPE-PEG(2000)-CREKA (100 μM PAMs) (Additional file 1: Table S1). No aggregation was observed by dynamic light scattering (DLS) for 0.05 μmol and 0.25 μmol Fe-NP, and diameters (< 20 nm) for hybrid nanoparticles were similar to that of CREKA-Ms without any Fe-NP. For 0.5 μmol Fe-NP samples, a small sediment was precipitated at the bottom of the solution, indicating 0.5 μmol Fe-NP is above the maximum loading capacity for 100 μM PAMs (Additional file 1: Fig. S4). Therefore, 5:1 molar ratio of Fe-NP and DSPE-PEG(2000)-CREKA was chosen as optimal and scaled up accordingly. Similar loading experiments were performed between 0.05 and 1 μmol Mn-NP for 100 μM PAMs, and 10:1 mol ratio Mn-NP and DSPE-PEG(2000)-CREKA was found as the optimal working ratio (Additional file 1: Table S2).

**Fig. 1:**
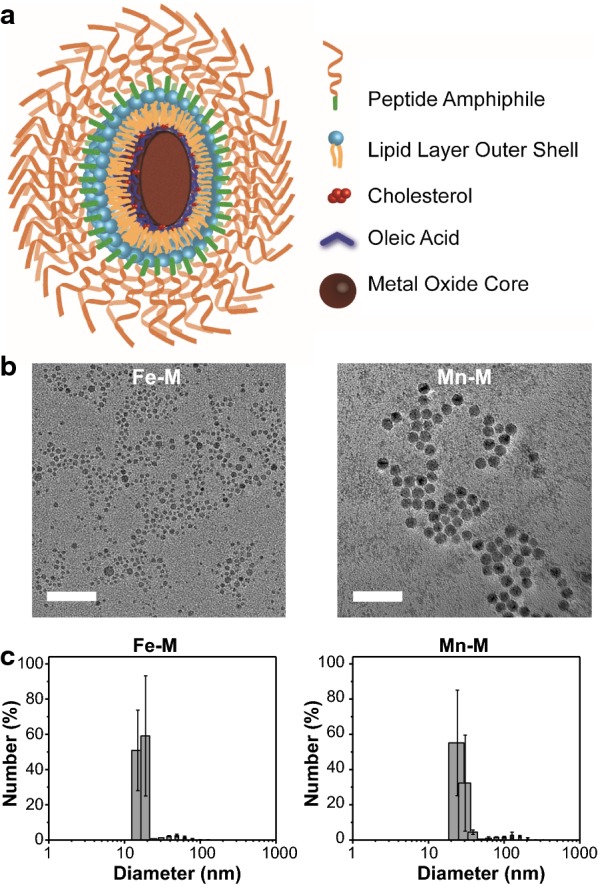
Preparation and characterization of HMO-Ms. **a** Schematic representation of HMO-Ms. **b** TEM images of Fe-Ms and Mn-Ms after phase transfer. Scale bar = 50 nm. **c** Particle size distribution of Fe-Ms and Mn-Ms determined by DLS

To further enhance the stability and permeability of HMO-Ms, 1,2-dioleoyl-*sn*-glycero-3-phosphocholine (DOPC) and cholesterol were incorporated together with DSPE-PEG(2000)-CREKA onto the oleate exterior of the metal oxide core to form an asymmetric bilayer. DOPC is commonly used on the surface of nanoparticles to mimic the lipid bilayer of cells, which enhances the interaction with cells [39, 40]. About 20–50 mol% of cholesterol is found in the phospholipid bilayer of mammalian cells, which provides order and stability to the membrane structure [41, 42]. In this study, 37 mol% cholesterol was used in our formulation of HMO-Ms. Driven by hydrophobic interactions, Fe-NP (5 molar) and Mn-NP (10 molar) were coated with DOPC, cholesterol, and DSPE-PEG(2000)-CREKA at 1.33:1.33:1 molar ratio (Fig. 1a). Encapsulation of Fe-NP and Mn-NP within the asymmetric bilayer yielded slightly larger particles than the Fe-NP and Mn-NP core, with diameters of 20.4 ± 7.9 nm and 33.1 ± 5.5 nm, respectively, and was similar to particles encapsulated with DSPE-PEG(2000)-CREKA only (Fig. 1b, c, Table 1 and Additional file 1: Table S3).

**Table 1:** Characterization of PAMs

	Diameter (nm)	PDI	Zeta potential (mV)
Fe-Ms	20.4 ± 7.9	0.151 ± 0.063	− 23.8 ± 2.3
NT-Fe-Ms	19.8 ± 2.4	0.112 ± 0.016	− 35.5 ± 0.7
Mn-Ms	33.1 ± 5.5	0.120 ± 0.056	− 22.9 ± 0.5
NT-Mn-Ms	38.0 ± 9.4	0.224 ± 0.096	− 26.7 ± 6.3

In order to evaluate the stability of HMO-Ms, the nanoparticles were exposed to BSA in PBS at 37 °C for 12 h (Additional file 1: Fig. S5). BSA bound to nanoparticles after 5 min, increasing the particle size to 65.9 ± 7.0 nm and 54.6 ± 13.9 nm for Fe-M and Mn-M, respectively. After initial BSA-binding, the particle size for both Fe-M and Mn-M remained stable throughout the 12 h incubation. In contrast, HMO-Ms without the inclusion of DOPC and cholesterol in the bilayer steadily increased in particle size between 2 and 4 h, indicating that DOPC and cholesterol is necessary to protect the nanoparticle against protein binding and aggregation.

The zeta potentials of Fe-Ms and Mn-Ms were − 23.8 ± 2.3 mV and − 22.9 ± 0.5 mV, respectively (Table 1). The positive increase in zeta potentials of Fe-Ms and Mn-Ms could be attributed to the presence of positively-charged arginine and lysine residues in the fibrin-binding peptide on the surface of the nanoparticles. Non-targeted HMO-Ms (NT-Fe-Ms and NT-Mn-Ms) were similarly synthesized using DSPE-PEG(2000)-methoxy, which served as a control for in vitro studies (Additional file 1: Table S4, Figs. S6, S7). NT-Fe-Ms and NT-Mn-Ms showed particle sizes of 19.8 ± 2.4 nm and 38.0 ± 9.4 nm, respectively, and a zeta potential of − 35.5 ± 0.7 mV and − 26.7 ± 6.3 mV, respectively.

### In vitro release profiles

**Fig. 2:**
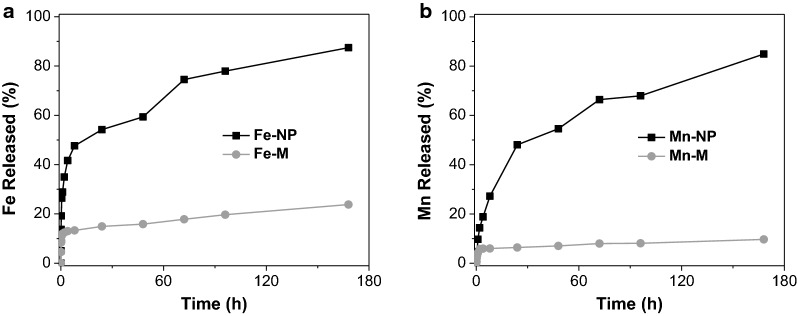
In vitro release profile of Fe and Mn from bare metal oxide nanoparticles and HMO-Ms show successful encapsulation of HMO-Ms. **a** Fe release profiles of Fe-Ms and **b** Mn release profiles of Mn-Ms in PBS buffer at 37 °C

In vitro release rates of Fe and Mn ions from HMO-Ms were investigated in PBS buffer at 37 °C in pH 7.4 to compare the stability between the bare metal oxide nanoparticles and HMO-Ms. The release kinetics of metal ions from HMO-Ms were much slower than that of the bare nanoparticle, thereby confirming the successful encapsulation of the metal oxide core within HMO-Ms (Fig. 2). Fe and Mn ion release was 24% and 10% for Fe-Ms and Mn-Ms, respectively, after 168 h, while the bare Fe-NP and Mn-NP particles showed rapid burst release, with 75% total Fe and 66% total Mn release before 72 h, thus demonstrating protection against degradation by the asymmetric bilayer.

### In vitro biocompatibility

**Fig. 3:**
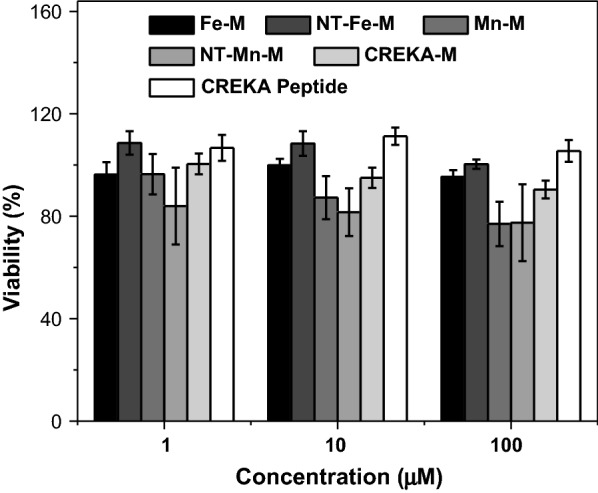
HMO-Ms are biocompatible in vitro. Viability of hAECs treated with Fe-Ms, NT-Fe-Ms, Mn-Ms, NT-Mn-Ms, CREKA-Ms, and CREKA peptides at varying CREKA concentrations for 72 h through an MTS assay (n = 6)

Since endothelial cell activation and dysfunction are essential for the formation of atherosclerotic plaque, in vitro biocompatibility assays of HMO-Ms were carried out against human aortic endothelial cells (hAECs) (Fig. 3) [43, 44]. Upon incubation of hAECs with micelle concentrations of 1, 10, and 100 μM (or 4.6, 46, and 460 μM Fe or 0.92, 9.2, and 92 mM Mn) for 72 h, MTS assays showed cells were over 90% viable for Fe-Ms, NT-Fe-Ms, CREKA-Ms, and free CREKA peptides as compared to the PBS control. No difference in viability was observed between Fe-Ms and CREKA-Ms, suggesting that the inclusion of the Fe-NP core to PAMs did not produce toxicity to hAECs. However, slight toxicity (> 75% viability) was seen with the inclusion of the Mn-NP core (Mn-M and NT-Mn-M) at 100 μM PAM concentration due to the cellular toxicity of Mn in ionic form [25, 45]. These results indicate that Fe-Ms are more biocompatible than Mn-Ms, while Mn-Ms were found to be non-toxic (> 90% viability) up to 10 μM.

### In vitro binding assay

**Fig. 4:**
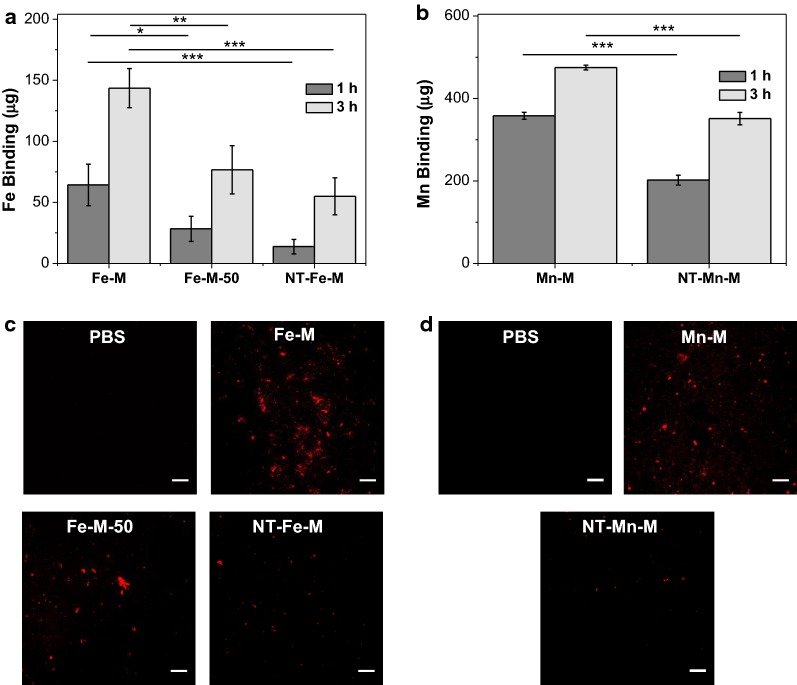
HMO-Ms demonstrate higher fibrin clot binding affinity. **a** Fe content of fibrin clots after 1 h and 3 h exposure to Fe-Ms. **b** Mn content of fibrin clots after 1 h and 3 h exposure to Mn-Ms (n = 4; *p < 0.05, **p < 0.01, ***p < 0.001). **c** Confocal microscopy images of Fe-Ms and **d** Cy5-Mn-Ms (red fluorescence) after 3 h of incubation with fibrin clots. Scale bar = 20 µm

As microthrombi and fibrin deposition are found on necrotic cores of plaque, the fibrin-targeting capability of HMO-Ms was determined by an in vitro clot-binding assay (Fig. 4). Following polymerization of human plasma and thrombin, the clot was incubated with PBS, HMO-Ms, or NT-HMO-Ms for 1 h or 3 h at 37 °C and evaluated for elemental analysis and imaging (Fig. 4a, b). Based on Fe content bound to the clot, Fe-Ms were found to have a 4.7-fold and 2.7-fold higher binding than NT-Fe-Ms at 1 h and 3 h, respectively. Notably, Fe-Ms continued to bind over time and showed a 2.2-fold increase in binding at 3 h compared to 1 h. When the CREKA targeting peptide was reduced by 50% on Fe-Ms (Fe-M-50), these nanoparticles had 2.3-fold less binding to human clots at 1 h incubation and 1.9-fold less binding at 3 h incubation than that of Fe-Ms. Similarly, Mn-Ms were found to have a 1.8-fold and 1.4-fold higher binding than its non-targeted counterpart at 1 h and 3 h, respectively, which demonstrates the targeting potency and specificity of the CREKA peptide.

To visualize the binding of HMO-Ms on fibrin clots, HMO-Ms were labeled with Cy5 and evaluated using confocal microscopy (Fig. 4c, d). A strong Cy5 fluorescence signal (red) of Fe-Ms was found on the fibrin clot compared to a weaker Cy5 signal observed for Fe-Ms-50, and minimal fluorescence was found for NT-Fe-Ms. Similarly, Mn-Ms demonstrated the same trend in which binding affinity was higher for Mn-Ms than for NT-Mn-Ms.

### *r*_1_ and *r*_2_ measurements of HMO-Ms

**Fig. 5:**
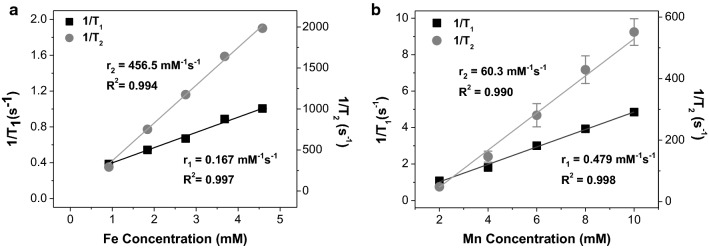
HMO-Ms exhibit enhanced MR relaxivities. Longitudinal (*r*_1_, black) and transverse (*r*_2_, gray) MR relaxivity plots of **a** Fe-M and **b** Mn-M

To characterize and quantify the MRI potential of HMO-Ms, MR relaxivities were determined using a 11.4 T NMR at varying concentrations of Fe-Ms and Mn-Ms (Fig. 5). The *T*_2_-weighted Fe-Ms were found to have a longitudinal (*r*_1_) and transverse relaxivities (*r*_2_) of 0.170 mM^−1^ s^−1^ and 456.5 mM^−1^ s^−1^, respectively (Fig. 5a). Notably, the *r*_2_ value from these Fe-Ms exceeded that of other reported and commercially available Fe nanoparticles in the literature (50–300 mM^−1^ s^−1^) (Table 2). Hence, the addition of CREKA PAs and the lipid bilayer did not alter the influence of the Fe centers on the surrounding water molecules. Additionally, Mn-Ms showed enhancement in longitudinal relaxivities (*T*_1_). Mn-Ms exhibited an *r*_1_ value of 0.479 mM^−1^ s^−1^ and an *r*_2_ value of 60.3 mM^−1^ s^−1^, which are comparable to other Mn-NPs (Fig. 5b, Table 3).

**Table 2:** Summary of MR relaxivities of iron oxide nanoparticles in the literature

Iron oxide nanoparticle	Diameter (nm)	*r*_1_ (mM^−1^ s^−1^)	*r*_2_ (mM^−1^ s^−1^)	Field (T)	References
MION	16	25	150	0.47	[47]
PVP-grafted MNP	14	2.6	72	1.5	[48]
USPION	20	5.1	94	1.4	[49]
LSPIO	39	11	119	9.4	[20]
USPION	11	7.4	218	7	[50]
USPION	35	6.6	128	3	[51]
USPION	35	15.5	100	1.5	[51]
Ferumoxtran-10	18	24	53	0.47	[52]
Feridex	120–180	23.9	98.3	0.47	[53]
Resovist	60	11	130	1.4	[54]
Feraheme	20–30	3.1	68	7	[55]
VSOP	4	15	32	1.5	[55]
ZES-SPION	4.4	1.5	17	7	[55]
*Fe-M*	*20.4*	*0.2*	*457*	*11.4*	

*MION* monocrystalline iron oxide nanoparticle, *PVP-grafted MNP* poly(*N*-vinyl pyrrolidone) functionalized magnetite nanoparticles, *USNP* ultrasmall superparamagnetic iron oxide nanoparticle, *LSPIO* lipid-coated ultrasmall superparamagnetic iron particles, *VSOP* very small iron oxide nanoparticle, *ZES-SPION* zwitterion-coated exceedingly small iron oxide nanoparticle

**Table 3:** Summary of MR relaxivities of manganese oxide nanoparticles in the literature

Manganese oxide nanoparticle	Diameter (nm)	r_1_ (mM^−1^ s^−1^)	r_2_ (mM^−1^ s^−1^)	Field (T)	References
MnO	7	0.37	1.74	3.0	[38]
MnO	15	0.18	0.57	3.0	[38]
MnO	20	0.13	0.52	3.0	[38]
MnO	25	0.12	0.44	3.0	[38]
MnO	10	0.81	–	3.0	[56]
Mn_3_O_4_	10	1.31	6.42	3.0	[57]
Mn_3_O_4_ hollow	20	1.42	7.74	1.5	[58]
MnO hollow	20	1.15	6.73	1.5	[59]
*Mn-M*	*33*	*0.48*	*60.3*	*11.4*	

### MRI using HMO-Ms on human clots

**Fig. 6:**
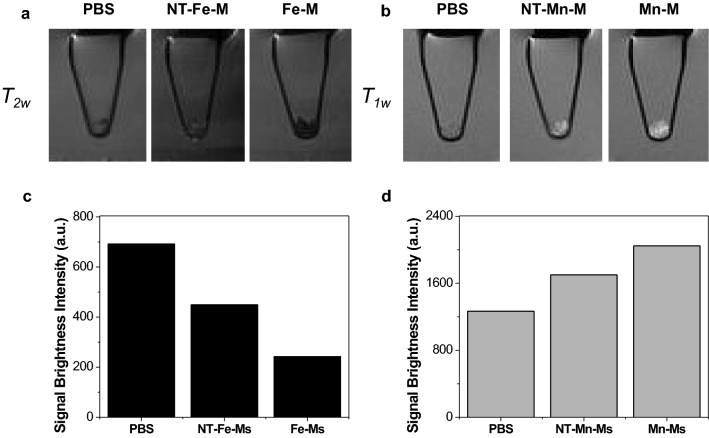
HMO-Ms produce MR signal enhancement of fibrin clots. **a** *T*_2_- and **b** *T*_1_- weighted images of fibrin clots after 3 h exposure to Fe-Ms and Mn-Ms, respectively. Signal brightness intensities showing enhanced binding of **c** Fe-Ms and **d** Mn-Ms

To validate the feasibility of HMO-Ms for MRI applications, the targeting capabilities of the nanoparticles on clots were tested through MR imaging (Fig. 6). Consistent with the *r*_1_ and *r*_2_ values, an enhancement in signal brightness (increase for *T*_1_ and decrease for *T*_2_) was determined for Fe-Ms- and Mn-Ms-bound clots, and was statistically significant compared to non-targeted nanostructures. Both Mn-Ms and Fe-Ms were able to modify the signal intensity of MR images in a similar manner: 62% for Mn-Ms and 65% for Fe-Ms versus 34% and 35% for NT-Mn-Ms and NT-Fe-Ms, respectively. The close performance of the HMO-Ms in MRI highlights the versatility of the methodology developed in this work. These results confirmed the ability of Fe-Ms and Mn-Ms to act as effective *T*_1_ and *T*_2_-weighted contrast agents, and both platforms have the potential to provide important diagnostic information in MR imaging of fibrin-containing diseases like atherosclerosis.

## Conclusions

In this study, we developed a hybrid nanoparticle platform that consists of a highly crystalline inorganic metal oxide core encapsulated within organic, fibrin-targeting PAs for enhanced binding to clots for applications in atherosclerosis. These hybrid nanoparticles were self-assembled and found to be monodispersed and spherical in shape with a diameter of ~ 20–30 nm. The DOPC and cholesterol of the asymmetric, exterior lipid bilayer of HMO-Ms provided additional stability as seen in the lack of particle aggregation upon serum incubation. In vitro, HMO-Ms were proven to be both non-toxic and biocompatible with hAECs. Moreover, HMO-Ms were found to be effective MRI contrast agents with high *r*_1_ and *r*_2_ relaxivities for Mn-M and Fe-M, respectively, and upon incubation with fibrin-containing clots, the CREKA targeting moiety provided enhanced clot specificity, with three to five times higher binding than its non-targeted particles for both Mn-Ms and Fe-Ms. Our hybrid nanoplatform offers potential for early diagnosis of thrombosis in atherosclerosis, and future studies will focus on the in vivo MRI capability in mice and large animal models carrying atherosclerotic plaques.

## Experimental methods

### General experimental

All starting materials were purchased from Sigma-Aldrich (St. Louis, MO, USA) and Fisher (Hampton, NH, USA), unless otherwise noted, and used without further purification. 1,2-distearoyl-*sn*-glycero-3-phosphoethanolamine-*N*-[amino(polyethylene glycol)2000] (DSPE-PEG(2000)) was purchased from Avanti Polar Lipids (USA). Human aortic endothelial cells (hAECs) were purchased from Lonza (Switzerland) and cultured in EBM-2 medium supplemented with 2% FBS and VEGF. All cell types were cultured in a humidified incubator at 37 °C under 5% CO_2_. Cells at passage five were used and media was changed every 2–3 days.

### Preparation of iron oxide nanoparticles (Fe-NPs)

A thermal decomposition method was followed to synthesize Fe_3_O_4_ nanoparticles. Briefly, oleic acid (6 mmol), oleylamine (6 mmol), 1,2-hexadecanediol (10 mmol) and Fe(acac)_3_ (2 mmol) were mixed into 20 mL of phenyl ether. The solution was heated under vigorous magnetic stirring, with reflux and Ar first at 200 °C for 30 min, and then for another 30 min at 265 °C. The resulting solution had a dark brown color. After cooling down to room temperature, 40 mL of ethanol were added into the solution to precipitate the magnetic nanoparticles. The nanoparticles were separated magnetically, washed 3 times with ethanol, and then resuspended in 75 mM oleic acid and 75 mM oleylamine solution of hexane until further use.

### Preparation of manganese oxide nanoparticles (Mn-NPs)

A two-steps protocol was followed for the preparation of manganese oxide nanoparticles. The first step was the synthesis of the manganese precursor: Sodium oleate (24.36 g) was mixed with manganese (II) chloride (7.92 g) in a solvent mixture 3:7:4 of ethanol:hexane:water (140 mL in total). This mixture was stirred at 70 °C overnight and was transferred to a separating funnel the next morning. The aqueous layer was discarded and the organic layer was washed three times with water. Finally, the organic phases were combined and evaporated to dryness to give a pink-red solid product of manganese oleate. To prepare MnO nanoparticles, manganese oleate (1.24 g) was dissolved in 1-hexadecene (10 g) in a 3-neck round-bottom flask with a reflux system. The solution was heated to 80 °C under vacuum to remove hexane-water. After 90 min, the vacuum was exchanged for N_2_ and the temperature was raised to 280 °C and maintained for 30 min. The dark solution was allowed to cool down to room temperature and acetone (10 mL) was added to precipitate the nanoparticles. The particles were then centrifuged and washed again with acetone. Finally, they were resuspended in 75 mM oleic acid hexane solution and stored in the fridge until further use.

### Synthesis of CREKA peptide

CREKA peptide was synthesized according to protocols in the literature [36]. Briefly, the peptide was synthesized on a Rink resin from C-terminus to N-terminus via the Fmoc/t-Bu strategy on an automated peptide synthesizer (PS3, Protein Technologies, Tucson, AZ, USA). Cysteine was added on the N-terminus for conjugation to DSPE-PEG(2000)-maleimide. A solution of 94:2:5:2:5:1 vol% TFA:1,2-ethanedithiol:water:triisopropylsilane was used to cleave the peptide off the resin. The resulting cleaved peptide was precipitated and washed twice with ice-cold diethyl ether. The crude peptide was purified by reverse-phase high performance liquid chromatography (HPLC) on a Luna C8 column (250 × 10 mm ID, 5 mm, Phenomenex, Torrance, CA, USA) using at 55 °C using 0.1% formic acid in acetonitrile/water mixtures and characterized by matrix-assisted laser desorption/ionization (MALDI) mass spectroscopy.

### Synthesis of DSPE-PEG(2000)-CREKA

The cysteine containing peptide was conjugated onto DSPE-PEG(2000)-maleimide to afford DSPE-PEG-peptide conjugate via thioether linkage. After 24 h at room temperature, the crude product was purified by HPLC and characterized by MALDI as described above. A fluorescent-labeled Cy5 DSPE-PEG(2000) conjugate was also synthesized using similar methods.

### Encapsulation of hybrid metal oxide peptide amphiphile micelles (HMO-Ms)

HMO-Ms were self-assembled by adding a chloroform solution of DOPC, cholesterol, and DSPE-PEG(2000)-CREKA (DOPC:cholesterol:amphiphiles = 1.33:1.33:1 molar ratio) with Fe-NP or Mn-NP. The chloroform solution was stirred and evaporated under nitrogen. The resulting film was dried overnight and hydrated at 80 °C for 30 min in water or phosphate buffered saline (PBS), and the HMO-M solution was allowed to cool to room temperature.

### Characterization of bare metal oxide nanoparticles and HMO-Ms

Bare metal oxide nanoparticles were imaged through high resolution transmission electron microscopy (HR-TEM, JEM-2100F, JEOL, Tokyo, Japan) at a working voltage of 200 kV. Energy-dispersive X-ray spectroscopy (EDXS, JEM-2100F, JEOL, Tokyo, Japan) was utilized to analyze the Fe and Mn concentration of Fe-NPs and Mn-NPs, respectively. The bare Fe-NP samples were diluted (1:1000) and applied onto a 400 mesh carbon-coated Cu grid (Agar Scientific, UK) followed by evaporation of solvent under vacuum overnight. Particle sizes and zeta potentials of 100 μM HMO-Ms were analyzed using DLS (Mobius, Wyatt, Santa Barbara, CA, USA). All DLS and zeta potential measurements were performed in three replicas and reported as mean ± standard deviation. To visualize the morphology of the nanoparticles, 100 μM PAM solutions were negatively stained with 2 wt% uranyl acetate solution on 400 mesh carbon grids (Ted Pella, Redding, CA, USA) and imaged using TEM (JEM-2100F, JEOL, Tokyo, Japan).

### Stability studies of HMO-Ms with and without DOPC and cholesterol

100 μM of Fe-Ms and Mn-Ms with and without DOPC and cholesterol were incubated with 5 mg/mL bovine serum albumin (BSA) at 37 °C in PBS. A baseline particle size measurement was detected using DLS prior to the addition of BSA. Particle size and distribution were measured every 2 h for 12 h to determine the particle stability in suspension over time.

### Release profiles of HMO-Ms

Elemental release of Fe and Mn from bare metal oxide nanoparticles and HMO-Ms were performed in 250 mL in PBS buffer at 37 °C in pH 7.4. In a 10,000 MWCO pleated dialysis bag (Fisher, St. Louis, MO, USA) Fe-Ms and Mn-Ms containing 0.92 μmol Fe or 2 μmol Mn were suspended in 2 mL PBS. At predetermined time points, 1 mL aliquots were taken from the PBS solution and replenished with fresh buffer solution. The removed aliquot was digested in concentrated nitric acid and analyzed for Fe or Mn via inductively coupled plasma optical emission spectrometry (ICP-OES, iCap 7400 Analyzer, ThermoFisher).

### In vitro biocompatibility

hAECs were plated in 96-well plates at a cell density of 2000 cells/well and varying concentrations of CREKA peptide, CREKA-Ms, Fe-Ms, and Mn-Ms in PBS were added to wells. The plates were incubated at 37 °C and 5% CO_2_ for 72 h, and cell viability was determined via MTS assay (BioVision, Milpitas, CA, USA) according to manufacturer’s instructions. Absorbance at 490 nm was used to measure the enzymatic activity level, and 100% viability using non-treated, healthy cells was used as a positive control. Viability of each treatment group was measured from six replicates.

### Fibrin clot binding

To evaluate the binding affinity of HMO-Ms, fibrin-containing clots were synthesized based on previous literature [46]. Briefly, human plasma (225 μL) was mixed with 75 μL of 100 mM CaCl_2_ solution. Thrombin (5 units at 0.5 unit/μL) was added to the plasma and stirred evenly. The fibrin clot was allowed to incubate at room temperature for 30 min or until it turned into a gel. PBS, bare metal oxide nanoparticles, or HMO-Ms at 100 μM PAM (or 457 μM Fe or 920 μM Mn) were added onto the fibrin clot and incubated for 1 h or 3 h at 37 °C. The clot was washed three times with PBS prior to analysis. For elemental analysis, the fibrin clot was digested with concentrated nitric acid and analyzed for Fe or Mn amount by ICP-OES. For confocal microscopy, the fibrin clot was incubated with Cy5-labeled PAMs (10 mol% Cy5 amphiphiles) for 3 h and fixed with 4% paraformaldehyde, prior to observation using confocal laser scanning microscopy (CLSM, Zeiss, Oberkochen, Germany) at excitation wavelengths of 650 nm to visualize the nanoparticle (red).

### *r*_1_ and *r*_2_ analysis

Using 500 MHz NMR at 37 °C (11.4 T, Varian), longitude (T_1_) and transverse (T_2_) relaxation rate was determined at six different concentration levels (0–4.6 mM Fe or 0–1000 μM PAMs and 0–10 mM Mn or 0–1000 μM PAMs). All relaxivity values were obtained as the sloped associated with the linear fit of Fe and Mn concentration, respectively, v. 1/*T*_1_ or 1/*T*_2_.

### MRI analysis on fibrin clot

For MR imaging the clots were transferred to PCR microtubes, centrifuged for 60 s at 10,000 rpm to ‘pellet’ the clots and then 200 μL of water were added into each tube. The microtubes were then mounted on a custom printed poly-(lactic acid) holder and imaged in a 3.0 T horizontal bore MR Solutions Benchtop MRI system equipped with 48 G/cm actively shielded gradients. To image the samples, a 56-mm diameter quadrature birdcage coil was used in transmit/receive mode. All MR images were acquired with an image matrix 256 × 252, FOV 60 × 60 mm, 2 sets of 3 slices each with a slice thickness of 1 mm. For *T*_2_-weighted imaging, fast spin echo (FSE) sequences with the following parameters were used: T_E_ = 68 ms, T_R_ = 4800 ms, N_A_ = 10, A_T_ = 24 m 53 s. For *T*_1_-weighted imaging, fast spin echo (FSE) sequences with the following parameters were used: T_E_ = 11 ms, T_R_ = 720 ms, N_A_ = 20, A_T_ = 15 m 41 s.

### Statistical analysis

For all experiments in this work, mean ± SD was calculated. A two-tailed Student *t*-tests and a one-way analysis of variance (ANOVA) was used to determine statistical significance between two groups and more than two groups, respectively. A *P* value < 0.05 was considered statistically significant.

## Additional file

**Additional file 1.** Additional figures and tables.

## Abbreviations

MRI: magnetic resonance imaging
HMO-Ms: hybrid metal oxide peptide amphiphile micelles
Fe-Ms: iron oxide peptide amphiphile micelles
Mn-Ms: manganese oxide peptide amphiphile micelles
hAECs: human aortic endothelial cells
CVD: cardiovascular disease
EPR: enhanced permeability and retention
Fe-NPs: iron oxide nanoparticles
Mn-NPs: manganese oxide nanoparticles
PAMs: peptide amphiphile micelles
TEM: transmission electron microscopy
PAs: peptide amphiphiles
DLS: dynamic light scattering
DOPC: 1,2-dioleoyl-*sn*-glycero-3-phosphocholine
NT-HMO-Ms: non-targeted HMO-Ms
