# Supplementary material for: Hybrid, metal oxide-peptide amphiphile micelles for molecular magnetic resonance imaging of atherosclerosis
Source: J Nanobiotechnology. 2018 Nov 15;16:92. doi: 10.1186/s12951-018-0420-8 (PMC6238287; doi:10.1186/s12951-018-0420-8)
Supplement: Supplementary file 1 — Additional file 1. Additional figures and tables. [file 12951_2018_420_MOESM1_ESM.docx]

**Additional Information for**

**Hybrid, Metal Oxide-Peptide Amphiphile Micelles for Molecular Magnetic Resonance Imaging of Atherosclerosis**

Christopher Poon^a^, Juan Gallo^b^, Johan Joo^a^, Timothy Chang^a^, Manuel Bañobre-López*^b^, Eun Ji Chung*^a,c,d,e,f^

^a^Department of Biomedical Engineering, University of Southern California, 1042 Downey Way, Los Angeles, California, 90089, United States

^b^Advanced (magnetic) Theranostic Nanostructures Lab, Nanomedicine Group, International Iberian Nanotechnology Laboratory, Avenida Mestre José Veiga, Braga, Portugal.

^c^Department of Materials Science and Chemical Engineering, University of Southern California, 925 Bloom Walk, Los Angeles, California, 90089, United States

^d^Eli and Edythe Broad Center for Regenerative Medicine and Stem Cell Research, Keck School of Medicine, University of Southern California, Los Angeles, CA, USA

^e^Division of Nephrology and Hypertension, Department of Medicine, Keck School of Medicine, University of Southern California, Los Angeles, CA, USA

^f^Norris Comprehensive Cancer Center, Keck School of Medicine, University of Southern California, Los Angeles, CA, USA

*Co-corresponding authors

Eun Ji Chung

Department of Biomedical Engineering

University of Southern California

1042 Downey Way, Los Angeles, CA 90089

Tel.: +1-213-740-2825

Fax: +1-213-821-3897

Email: [eunchung@usc.edu](mailto:eunchung@usc.edu)

Email Addresses of Co-authors:

Christopher Poon ([poonc@usc.edu](mailto:poonc@usc.edu))

Juan Gallo ([juan.gallo@inl.int](mailto:juan.gallo@inl.int))

Johan Joo ([johanjoo@usc.edu](mailto:johanjoo@usc.edu))

Timothy Chang ([changtj@usc.edu](mailto:changtj@usc.edu))

Manuel Bañobre-López ([manuel.banobre@inl.int](mailto:manuel.banobre@inl.int))

**Additional Figures**


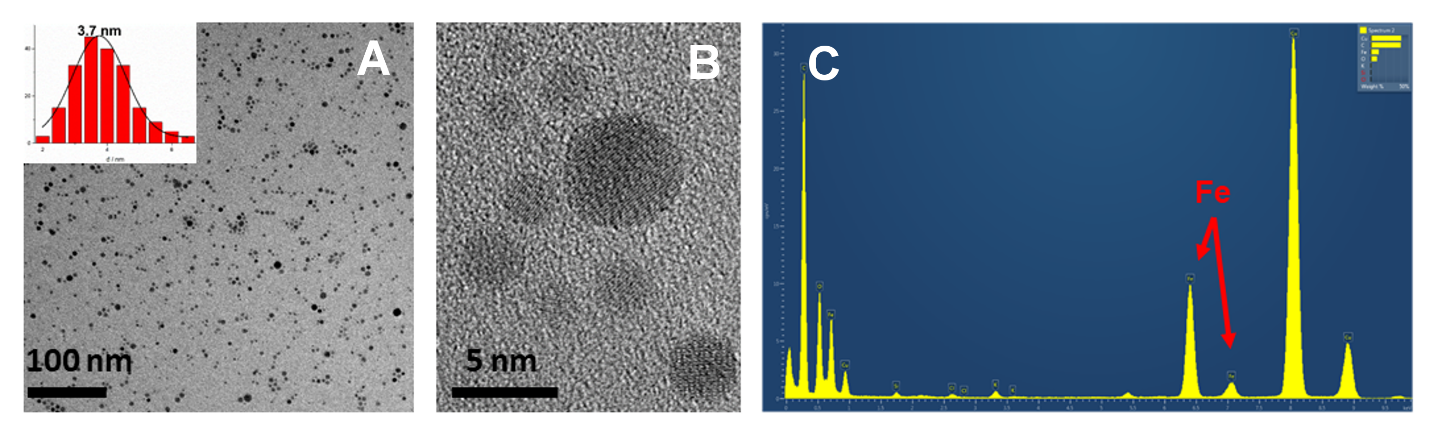


**Figure S1. (A)** TEM of iron oxide nanoparticles. Inset: Size distribution from TEM images. **(B)** HRTEM image of Fe-NPs showing the high crystallinity of the particle. **(C)** EDX spectra obtained from the sample in A and B show clear peaks from Fe (red arrows).


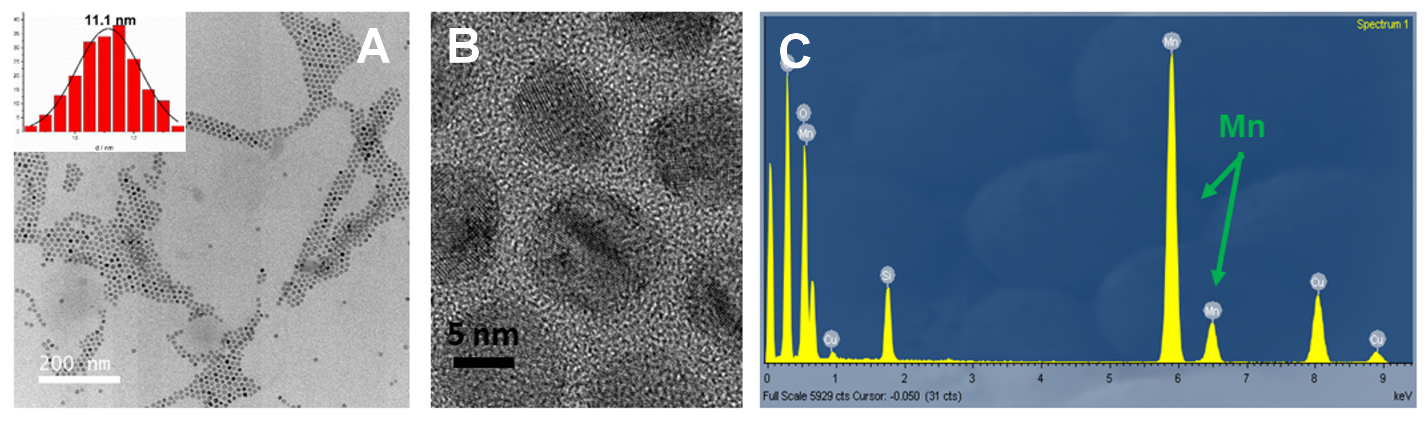


**Figure S2. (A)** TEM of manganese oxide nanoparticles. Inset: Size distribution from TEM images. **(B)** HRTEM image of Mn-NPs showing the high crystallinity of the particle. **(C)** EDX spectra obtained from the sample in A and B show clear peaks from Mn (green arrows).

**Figure S3.** HPLC chromatograms and MALDI-TOF mass spectra of **(A, B)** CREKA peptide and **(C, D)** DSPE-PEG(2000)-CREKA.


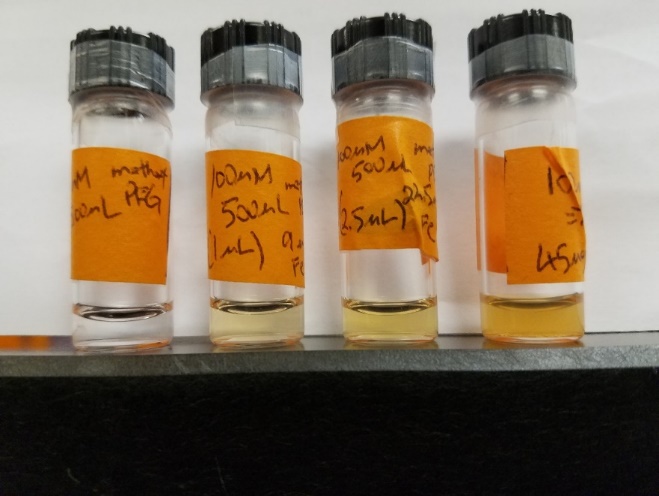


**Figure S4.** Iron oxide nanoparticles at 0, 0.05, 0.25, and 0.5 μmol (from left to right) containing 50 nmol of DSPE-PEG(2000)-methoxy in water solution.

**Figure S5.** The incorporation of DOPC and cholesterol stabilizes HMO-Ms from degradation. Particle size and distribution of **(A, B)** Fe-M and **(C, D)** Mn-M with (gray circle) and without (black square) DOPC and cholesterol when incubated with 5 mg/mL BSA in PBS for 12 h at 37^o^C.

**Figure S6.** Particle size distribution of **(A)** NT-Fe-Ms and **(B)** NT-Mn-Ms determined by DLS.

**Figure S7.** TEM images of **(A)** NT-Fe-Ms and **(B)** NT-Mn-Ms. Bar = 50 nm.

**Additional Tables**

**Table S1.** Particle size with varying amounts of iron oxide nanoparticles.

| Fe (μmol) | DSPE-PEG(2000)-methoxy (nmol) | Diameter (nm) | PDI |
| --- | --- | --- | --- |
| 0 | 50 | 22.4 ± 5.1 | 0.115 ± 0.001 |
| 0.05 | 50 | 14.3 ± 4.4 | 0.138 ± 0.026 |
| 0.25 | 50 | 26.7 ± 3.9 | 0.195 ± 0.033 |
| 0.5 | 50 | 126.0 ± 22.6 | 0.217 ± 0.052 |

**Table S2.** Particle characterization of varying amount of manganese oxide nanoparticles.

| DOPC (nmol) | Chol (nmol) | DSPE-PEG(2000)-CREKA(nmol) | Mn (μmol) | Diameter (nm) | PDI | Zeta Potential (mV) |
| --- | --- | --- | --- | --- | --- | --- |
| 67 | 67 | 50 | 0.05 | 46.3 ± 7.3 | 0.226 ± 0.097 | 22.0 ± 0.4 |
| 67 | 67 | 50 | 0.1 | 143.5 ± 3.1 | 0.261 ± 0.024 | 25.7 ± 2.5 |
| 67 | 67 | 50 | 0.5 | 33.1 ± 5.5 | 0.120 ± 0.056 | 22.9 ± 0.5 |
| 67 | 67 | 50 | 1 | 139.8 ± 5.2 | 0.242 ± 0.045 | 18.5 ± 0.3 |

**Table S3.** Particle size with and without DOPC and cholesterol in HMO-Ms.

| DOPC (nmol) | Chol (nmol) | DSPE-PEG(2000)-methoxy (nmol) | Mn (μmol) | Diameter (nm) | PDI |
| --- | --- | --- | --- | --- | --- |
| 67 | 67 | 50 | 0.5 | 26.7 ± 3.9 | 0.195 ± 0.033 |
| 0 | 0 | 50 | 0.5 | 20.1 ± 1.9 | 0.175 ± 0.049 |

**Table S4.** Particle size and zeta potential of NT-Fe-M and NT-Mn-M.

|  | Diameter (nm) | PDI | Zeta Potential (mV) |
| --- | --- | --- | --- |
| NT-Fe-Ms | 19.8 ± 2.4 | 0.112 ± 0.016 | -35.5 ± 0.7 |
| NT-Mn-Ms | 38.0 ± 9.4 | 0.224 ± 0.096 | -26.7 ± 6.3 |
